# Rapamycin Alleviates 2,3,7,8-Tetrachlorodibenzo-p-dioxin-Induced Aggravated Dermatitis in Mice with Imiquimod-Induced Psoriasis-Like Dermatitis by Inducing Autophagy

**DOI:** 10.3390/ijms22083968

**Published:** 2021-04-12

**Authors:** Hye Ran Kim, Jin Cheol Kim, Seok Young Kang, Hye One Kim, Chun Wook Park, Bo Young Chung

**Affiliations:** Department of Dermatology, Kangnam Sacred Heart Hospital, College of Medicine, Hallym University, Seoul 07441, Korea; cyberkhr@hanmail.net (H.R.K.); aiekfne@naver.com (J.C.K.); tjdjrdud@naver.com (S.Y.K.); hyeonekim@gmail.com (H.O.K.); dermap@daum.net (C.W.P.)

**Keywords:** rapamycin, 2,3,7,8-tetrachlorodibenzo-p-dioxin, autophagy, aryl hydrocarbon receptor, psoriasis

## Abstract

Recently, the mTOR signaling has emerged as an important player in the pathogenesis of psoriasis. We previously found that 2,3,7,8-tetrachlorodibenzo-p-dioxin (TCDD)-induced psoriatic skin inflammation was related to the inhibition of autophagy in keratinocytes. However, the effects and detailed molecular mechanisms of the mTOR inhibitor rapamycin and TCDD on psoriasis in vivo remain to be elucidated. In this study, we aimed to evaluate the effects of rapamycin and TCDD on skin lesions in imiquimod (IMQ)-induced psoriasis using a mouse model. TCDD aggravated skin inflammation in an IMQ-induced psoriatic mouse model. Furthermore, TCDD increased the expression of aryl hydrocarbon receptor (AHR), CYP1A1, proinflammatory cytokines, oxidative stress markers (NADPH oxidase (Nox) 2, Nox4), and phosphorylated P65NF-ĸB, whereas the expression of autophagy-related factors and the antioxidant marker nuclear factor-erythroid 2-related factor 2 (NRF2) decreased. Rapamycin reduced the aggravated skin inflammation induced by TCDD and restored TCDD-induced autophagy suppression and the increase of AHR expression, oxidative stress, and inflammatory response in the skin lesions of a psoriatic mouse model. In conclusion, we demonstrated that rapamycin alleviates TCDD-induced aggravated dermatitis in mice with imiquimod-induced psoriasis-like dermatitis through AHR and autophagy modulation.

## 1. Introduction

Psoriasis is a common chronic inflammatory skin disease that affects approximately 1–3% of the population worldwide [1]. This disease is clinically characterized by well-demarcated erythematous scaly plaques at distinct sites or disseminated on the whole body [2]. Histopathologic findings in lesional skin have shown epidermal hyperplasia, vessel dilatation, and dermal immune cell infiltrations [2]. Inflammation in psoriasis affects the skin and systemic circulation [3]. Psoriasis is a systemic inflammatory disease with a growing body of comorbidities, ranging from respiratory to cardiovascular and gastrointestinal diseases [4,5,6,7]. Despite the multiple pathogenetic mechanisms suggested in the literature, a detailed explanation of psoriasis-related comorbidities remains widely unknown. Due to the pleiotropic effect of mTOR in different tissues and the autophagic effect recently described [8,9], it could be a new research line to further comprehend psoriasis-related endotypes. Psoriasis is a multifactorial disease caused by immune dysfunction, genetic factors, and environmental factors [10,11]. Besides pollution, the inter-relation between concurrent inflammatory diseases such as asthma and psoriasis or liver diseases and psoriasis has been modelled [12,13].

Recently, the detrimental effects of environmental pollution on psoriasis have been suggested [14,15]. Emerging data have found that an environmental pollutant, such as urban dust and diesel exhaust regulate, Th17 differentiation and the IL-17 signaling pathway [14,16,17]. It has been suggested that the effect is mediated mainly by polycyclic aromatic hydrocarbons (e.g., 2,3,7,8-tetrachlorodibenzo-p-dioxin (TCDD)) via aryl hydrocarbon receptor (AHR) activation. IL-17 is well known as the main actor in the pathogenesis of psoriasis [18]. TCDD is produced by uncontrolled waste incinerators and is also a byproduct of chemical synthesis, including chlorinated herbicides and fungicides [19]. Due to its lipophilicity, TCDD can readily penetrate animal tissues and accumulate in the food chain. Thus, the primary source of human exposure to TCDD is animal-derived food products [20]. However, as the contaminated ambient air contains TCDD [21], air pollution and TCDD exposure are also related. However, the effects of TCDD on in vivo skin remain unclear.

Rapamycin is an inhibitor of mTOR (mechanistic target of rapamycin) complex 1 (mTORC1) [22]. mTORC1 signaling is associated with controlling autophagy [23]. In in vitro studies, rapamycin has been commonly used as an autophagy inducer [24]. Rapamycin is known for its immunosuppressive properties and antiproliferative properties in human organs, including the skin [25]. Evidence that autophagy dysfunction in keratinocytes could contribute to the pathogenesis of psoriasis has been presented [26,27]. mTOR inhibitor topicals are already used for psoriasis treatment in corticosteroid-sensitive areas, such as genitals and face [28]. However, there have been limited studies regarding the efficacy of systemic or topical rapamycin for psoriasis.

Thus, we explored the effects of topical rapamycin or TCDD on skin lesions in an imiquimod-induced psoriasis in an in vivo mouse model and the discrete action mechanisms of rapamycin and TCDD on skin inflammation.

## 2. Results

### 2.1. TCDD Aggravated Skin Inflammation, Whereas Rapamycin Restored Skin Inflammation in Imiquimod-Induced Psoriatic Dermatitis in Mice

**Figure 1:**
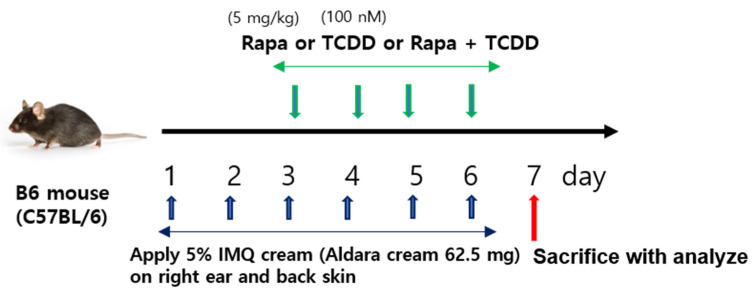
Study procedures. C57BL/6 mice were treated on the dorsal skin and right ear pinna for 4 days with five different treatment regimens as described in the Section 4. C57BL/6 mice were treated daily with vehicle control, IMQ, IMQ + TCDD, IMQ + Rapa, or IMQ + TCDD + Rapa applied on their shaved back skin and right ears. IMQ: imiquimod, Rapa: rapamycin.

**Figure 2:**
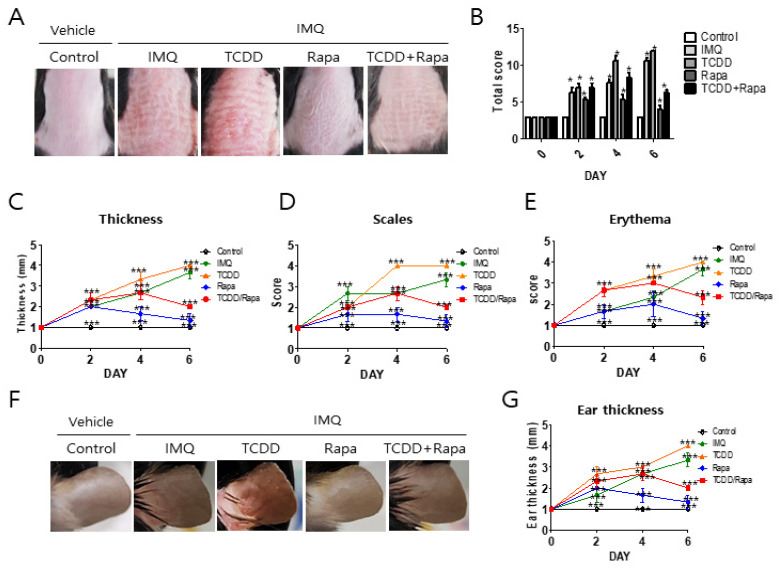
The effects of TCDD, rapamycin, and cotreatment with both on the clinical severity of imiquimod-induced psoriatic mice. C57BL/6 mice were treated daily with vehicle control, IMQ, IMQ + TCDD, IMQ + Rapa, or IMQ + TCDD + Rapa applied on their shaved back skin and right ears (*n* = 5 per treatment group). (**A**) Macroscopic presentation of dorsal skin of mice from the 5 treatment regimens. (**B**) The severity of inflammation on the back was assessed using a scoring system similar to the human Psoriasis Area and Severity Index (PASI) score. Thickness, scaling, and erythema of the back skin were scored “blindly” on a scale from 0 to 3 as follows: 0: none; 1: slight; 2: moderate; and 3: severe. (**C**–**E**) Detailed clinical disease score of thickness, scales, and erythema. Data represent mean ± standard deviation (SD). Statistical significance was determined by two-way repeated measures analysis of variance (ANOVA) with Tukey’s test. * *p* < 0.05; *** *p* < 0.001 compared with controls. (**F**) Macroscopic presentation of the right ear of mice from the 5 treatment regimens. (**G**) Assessment of ear skin thickness of the mice measured throughout the experiment. Statistical significance was determined by two-way repeated measures analysis of variance (ANOVA) with Tukey’s test. * *p* < 0.05; *** *p* < 0.001 compared with controls. IMQ: imiquimod, Rapa: rapamycin.

The efficacy of topical rapamycin or TCDD or cotreatment of rapamycin and TCDD in IMQ-induced psoriasis-like skin lesions was evaluated in the mouse model as shown in the study plan (Figure 1). IMQ cream or vehicle was applied to the shaved back skin and right ear pinna of C57BL/6 mice for 6 consecutive days. Two days after the start of IMQ application, the back skin and right ear of the mice started to display signs of erythema, scales, and swelling. The severity of psoriasis-like skin symptoms in IMQ-induced mice progressively increased until the end of the treatment (day 6). After 2 days of IMQ cream application, TCDD, rapamycin, or TCDD + rapamycin cream was applied in the morning for 4 days. Topical application of TCDD led to an increase in the severity of total cumulative scores as well as individual scores at 4 and 6 days compared with the IMQ group. Rapamycin treatment reduced the single and total scores on days 4 and 6 compared with the IMQ group. Compared with the TCDD-treated group, the combined TCDD and rapamycin treatment group showed significantly reduced individual scores and total scores of skin inflammation at days 2 and 4 (Figure 2A–E).

Regarding ear thickness, throughout the study period, IMQ induced a sequential increase. TCDD application led to a marked elevation in the thickness of the ear in IMQ-induced psoriatic mice compared with the IMQ-only-treated group at 2 and 4 days. Rapamycin restored the thickness of the ear in IMQ-induced psoriatic mice compared with the IMQ-only-treated group at 2 and 4 days. Furthermore, cotreatment with TCDD and rapamycin reduced ear thickness in IMQ-induced psoriatic mice compared with the TCDD-treated group. Taken together, rapamycin treatment resulted in significant improvement in the psoriasis-like skin phenotype induced by IMQ and restoration of aggravated skin inflammation by TCDD in IMQ-induced psoriatic mice.

### 2.2. Histopathological Changes with TCDD and Rapamycin Treatment in Imiquimod-Induced Psoriatic Mice

**Figure 3:**
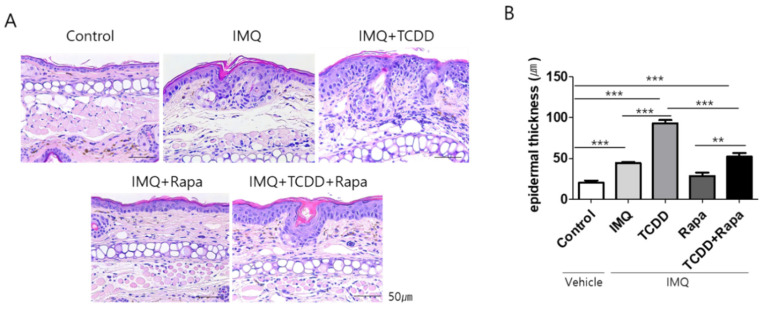
Histological changes after treatment with TCDD, rapamycin, or both in an IMQ-induced psoriasis mouse model. (**A**) Hematoxylin and eosin (H&E) staining of representative images from each group (dorsal skin). Bars represent 50 μm. (**B**) Evaluation of epidermal thickness in dorsal skin. The epidermal thickness was measured from the bottom of the rete ridge to the bottom of the stratum corneum; the mean was calculated from the measurements in four fields at ×100 magnification. Data represent mean ± standard deviation (SD). Statistical significance was determined by one-way repeated measures analysis of variance (ANOVA) with Dunnett’s test. ** *p* < 0.01; *** *p* < 0.001. IMQ: imiquimod, Rapa: rapamycin.

In hematoxylin and eosin (H&E)-stained sections of dorsal skin, IMQ induced marked acanthosis, hyperkeratosis, elongated rete ridges in the epidermis, and inflammatory cell infiltration in the dermis. TCDD showed more marked epidermal hyperplasia and inflammatory cell infiltration in the dermis in IMQ-induced psoriatic dermatitis lesions. Rapamycin treatment led to reduced IMQ-induced and TCDD + IMQ-induced histopathological changes (Figure 3A). Epidermal thickness was significantly increased in IMQ-treated mouse skin compared with control skin. TCDD induced a greater increase in epidermal thickness than IMQ only. Cotreatment with TCDD and rapamycin resulted in a significant reduction in epidermal thickness compared with TCDD-treated psoriatic skin (Figure 3B).

### 2.3. Effect of TCDD and Rapamycin Treatment on Spleen Size and Weight

**Figure 4:**
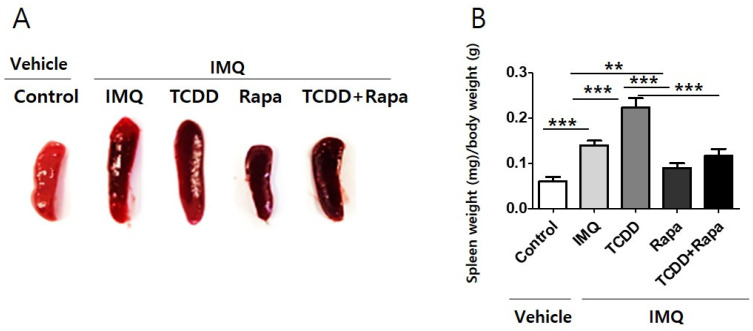
The spleen size and weight of mice from 5 study groups. (**A**) Spleens were prepared from each mouse and weighed. The spleen shown is from 1 representative experiment with *n* = 5 per treatment group. (**B**) The spleens from mice were isolated and weighed, and the spleen weight index was calculated as organ weight (milligram, mg) per gram (g) of mouse body weight. All data are presented as mean ± standard deviation (SD). Statistical differences were determined by one-way repeated measures analysis of variance (ANOVA) with Dunnett’s test. ** *p* < 0.01; *** *p* < 0.001. IMQ: imiquimod, Rapa: rapamycin.

We found a significant increase of spleen size and weight through induction of psoriasis in mice using IMQ. TCDD application led to an increase in spleen size and weight in IMQ-induced psoriatic mice. In contrast, rapamycin reduced spleen size and weight in IMQ-induced psoriatic mice and TCDD-treated IMQ-induced psoriatic mice. Cotreatment with TCDD and rapamycin yielded a reduction in spleen size and weight compared with TCDD treatment in psoriatic mice (Figure 4).

### 2.4. The Effects of TCDD and Rapamycin on the Expression of AHR and Autophagy-Related Factors

**Figure 5:**
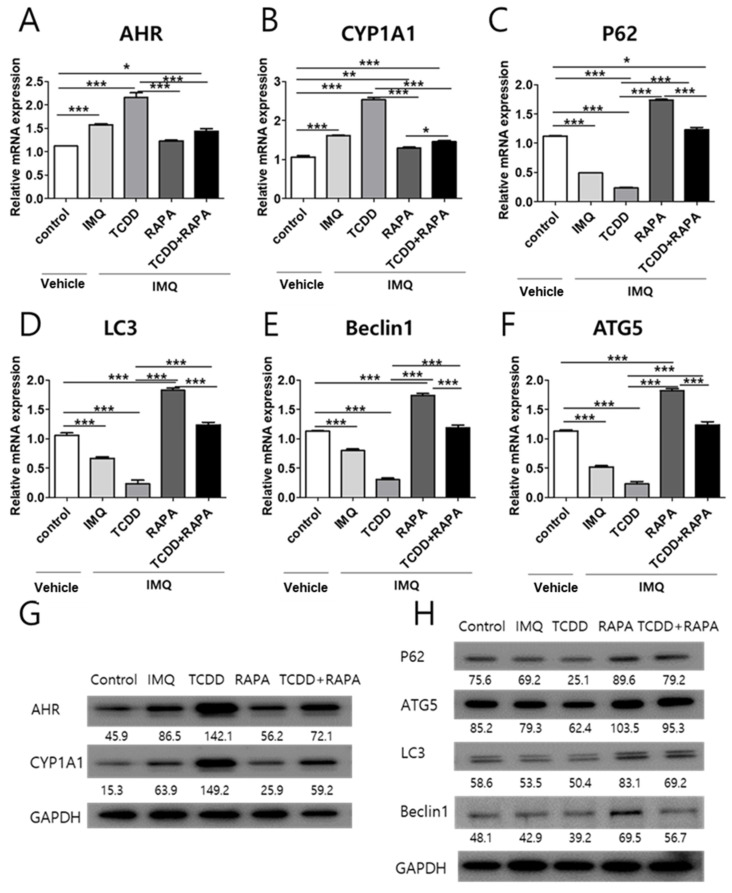
mRNA and Western blot analysis of AHR, CYP1A1, and autophagy-related factor expression changes in skin tissue with treatment with vehicle control, IMQ, IMQ + TCDD, and IMQ + Rapa, or IMQ + TCDD + Rapa. RNA and protein were extracted from the back skin. (**A**–**F**) qPCR and (**G**,**H**) Western blot analysis of AHR, CYP1A1, P62, ATG5, LC3, and Beclin1 expressions in mice treated with 5 different regimens (*n* = 5 per treatment group). qPCR data represent the mean ± standard deviation (SD) of three independent experiments (each performed in duplicate). Statistical differences were determined by one-way analysis of variance (ANOVA) followed by post hoc Dunnett’s test. * *p* < 0.05; ** *p* < 0.01; *** *p* < 0.001. Western blot normalization was based on GAPDH. Western blot data are representative of three independent experiments. IMQ: imiquimod, Rapa: rapamycin.

Next, we assessed whether TCDD and rapamycin affected the levels of AHR, CYP1A1, or autophagy-related factors, such as P62, ATG5, LC3, and Beclin1 mRNA or proteins, in psoriatic mouse skin. IMQ induced the elevation of AHR and CYP1A1 mRNA expression, whereas IMQ lowered the expression of P62, ATG5, LC3, and Beclin1. TCDD magnified the IMQ-induced change in the mRNA expression of AHR, CYP1A1, and autophagy-related factors. Cotreatment with TCDD and rapamycin reduced the mRNA expression of AHR and CYP1A1 and increased the mRNA expression of autophagy-related factors in IMQ-treated psoriatic mouse skin compared with the TCDD-treated group (Figure 5A–F). In Western blot analysis, AHR, CYP1A1, P62, ATG5, LC3, and Beclin1 protein levels also showed changes similar to that noted for mRNA expression with treatment with vehicle control, IMQ, IMQ + TCDD, and IMQ + Rapa, or IMQ + TCDD + Rapa (Figure 5G,H).

**Figure 6:**
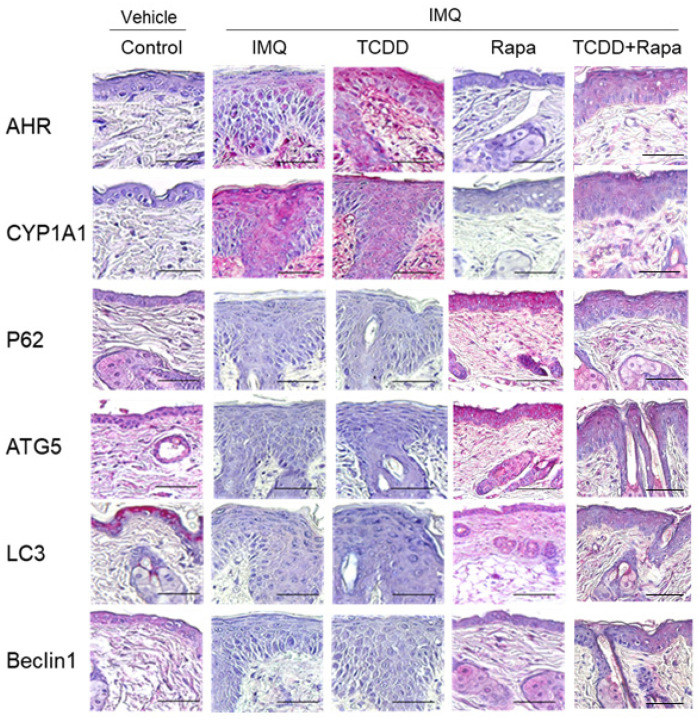
Immunohistochemical analysis of AHR, CYP1A1, and autophagy-related factors in skin tissue of mice from 5 different treatment regimens with vehicle control, IMQ, IMQ + TCDD, and IMQ + Rapa, or IMQ + TCDD + Rapa. Immunohistochemical staining with the indicated antibodies is shown from 1 representative mouse from each group (*n* = 5). Scale bars represent 50 μm. IMQ: imiquimod, Rapa: rapamycin.

Immunohistochemical analysis of AHR and autophagy-related factors in skin lesion tissue was obtained from imiquimod-induced psoriasis lesions of C57BL/6 mice treated with TCDD, rapamycin, and TCDD + rapamycin for 4 days (Figure 6). AHR and CYP1A1 expression levels were increased in the epidermis of IMQ-induced psoriatic mice compared with the control group. AHR expression levels were increased in the TCDD-treated IMQ-induced psoriatic mouse group compared with the IMQ-only-treated group. In the rapamycin-treated group, AHR and CYP1A1 expression levels were negative in the epidermis, which was similar to that noted in the control. In the TCDD + rapamycin cotreatment group, AHR and CYP1A1 expression levels in the epidermis were decreased compared with those in the TCDD-treated group. The expression levels of the autophagy-related factors P62, ATG5, LC3, and Beclin1 were positive in the epidermis of the lesional skin of rapamycin-treated psoriatic mice and control mice. In contrast, the expression levels of the autophagy-related factors P62, ATG5, LC3, and Beclin1 were negative in the epidermis of the IMQ-treated group and the TCDD-treated group. In the TCDD + rapamycin cotreatment group, P62, ATG5, LC3, and Beclin1 expression levels in the epidermis were decreased compared with those in the rapamycin-treated group.

### 2.5. TCDD Resulted in an Increase in the Expression of Proinflammatory Cytokines, Whereas Rapamycin Reduced the Expression of Proinflammatory Cytokines

**Figure 7:**
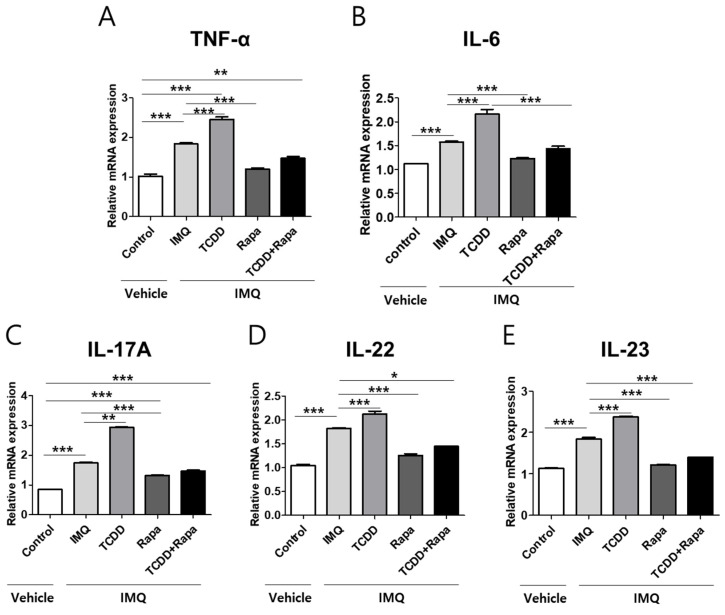
The effects of TCDD, rapamycin, or cotreatment of both on proinflammatory cytokine mRNA expression changes in the skin tissues of a psoriasis mouse model. (**A**–**E**) qPCR analysis of TNF-α, IL-6, IL-17A, IL-22, and IL-23 was performed in skin tissue from 5 different study groups (*n* = 5 per group). Data represent mean ± SD of three independent experiments (each performed in duplicate). Statistical differences were determined by one-way analysis of variance (ANOVA) with Dunnett’s test. * *p* < 0.05; ** *p* < 0.01; *** *p* < 0.001. Rapa: rapamycin; IMQ: imiquimod.

To evaluate the influence of TCDD, rapamycin, or combined TCDD and rapamycin on proinflammatory cytokines associated with the pathogenesis of psoriasis in vivo, TNF-α, IL-6, IL-17A, IL-22, and IL-23 mRNA expressions were assessed using qPCR in mouse skin tissue from five different treatment groups (control, IMQ, IMQ + TCDD, IMQ + rapamycin, or IMQ + TCDD + rapamycin) (Figure 7A–E). After IMQ treatment, TNF-α, IL-6, IL-17A, IL-22, and IL-23 mRNA expressions increased. Topical TCDD treatment resulted in a greater increase in TNF-α, IL-6, IL-17A, IL-22, and IL-23 mRNA expressions in IMQ-induced psoriatic dermatitis lesions in mice. Rapamycin reduced the expressions of these proinflammatory cytokines in IMQ-induced psoriatic dermatitis lesions. Furthermore, in the combined treatment with the TCDD and rapamycin group, rapamycin suppressed the expression of these proinflammatory cytokines in the psoriatic skin lesions of mice compared with the TCDD-treated group.

### 2.6. The Effects of TCDD and Rapamycin on the Expression of Oxidative Stress-Related Factors

**Figure 8:**
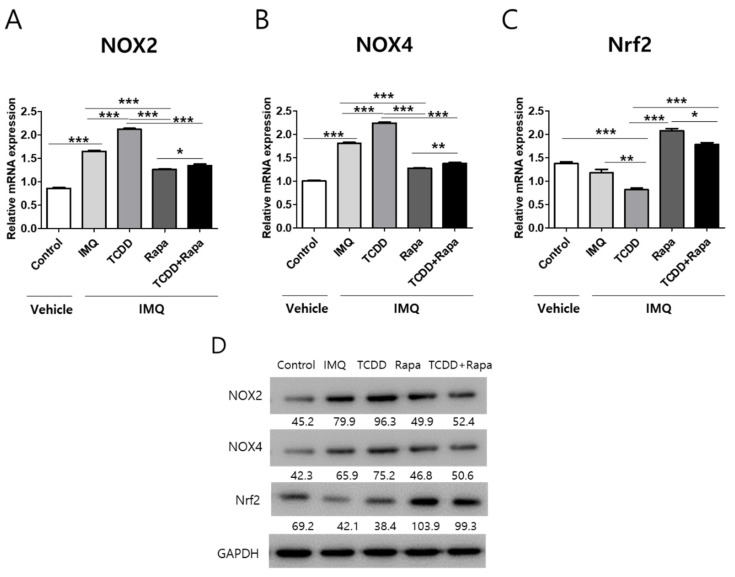
The effects of TCDD and rapamycin on the expression of oxidative stress-related factors. mRNA and protein expressions change in mouse skin tissue after treatment with vehicle control, IMQ, IMQ + TCDD, and IMQ + Rapa, or IMQ + TCDD + Rapa. (**A**–**C**) qPCR and (**D**) Western blot analyses of NOX2, NOX4, and Nrf2 expressions in mice treated with 5 different regimens (*n* = 5 per group). qPCR data represent the mean ± SD of three independent experiments (each performed in duplicate). Statistical differences were determined by one-way analysis of variance (ANOVA) with Dunnett’s post hoc test. * *p* < 0.05; ** *p* < 0.01; *** *p* < 0.001. Western blot normalization was based on GAPDH. Western blot data are representative of three independent experiments. Rapa: rapamycin; IMQ: imiquimod.

Next, we assessed whether TCDD and rapamycin affect the expression of oxidant and antioxidant signaling-related factors, such as NADPH oxidase (NOX) 2, NOX4, and nuclear factor-erythroid 2-related factor 2 (Nrf2). Nox2 and Nox4 are enzymes that generate reactive oxygen species [29], whereas Nrf2 is a transcription factor that controls the production of various antioxidative enzymes [30]. NOX2 and NOX4 mRNA expressions were upregulated by IMQ application in mouse skin. Topical TCDD application induced an increase in NOX2 and NOX4 mRNA expressions and a decrease in Nrf2 expression compared with the IMQ-only group in the psoriatic mouse model. Rapamycin suppressed the TCDD-induced upregulation of NOX2 and NOX4 expressions and increased the TCDD-derived downregulation of Nrf2 expression in psoriatic mouse lesional skin (Figure 8A–C). Western blot analysis showed that the effects of TCDD and rapamycin on NOX2, NOX4, and Nrf2 expression were similar to the mRNA expression changes.

### 2.7. The Effects of TCDD and Rapamycin on the NF-κB Signaling Pathway in Psoriatic Mouse Skin Lesions

**Figure 9:**
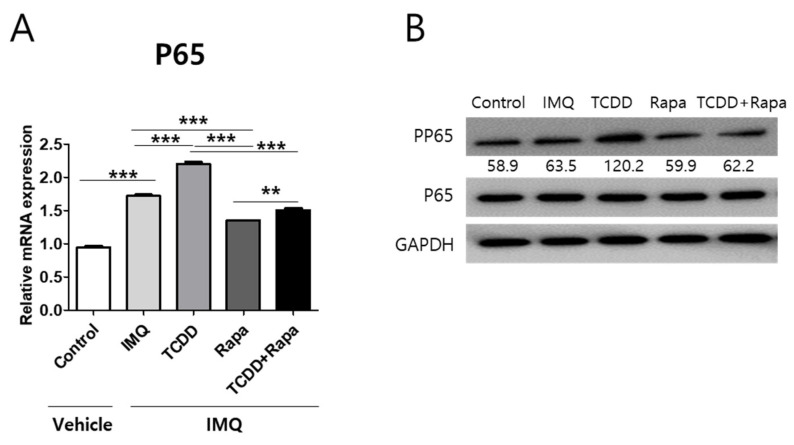
The effects of TCDD, rapamycin, or a combination of both on P65 mRNA and phosphorylation of P65 expression changes in mouse skin tissue. (**A**) qPCR of P65 and (**B**) Western blot analysis of phosphorylation of P65 expression in mouse skin from 5 different treatment regimens (*n* = 5 per group). Data represent the mean ± SD of three independent experiments (each performed in duplicate). Statistical differences were determined by one-way analysis of variance (ANOVA) followed by post hoc Dunnett’s test. ** *p* < 0.01; *** *p* < 0.001. Western blot normalization was based on GAPDH. Western blot data are representative of three independent experiments. IMQ: imiquimod, Rapa: rapamycin.

To identify whether the effects of TCDD and rapamycin on psoriatic skin lesions are related to NF-κB signaling, we assessed the influence of TCDD and rapamycin on P65/NF-κB mRNA expression and P65/NF-κB phosphorylation. In IMQ-induced mouse model lesional skin, P65 mRNA expression and P65 protein phosphorylation were increased compared with the control. TCDD aggravated the increase in these expression levels in IMQ-induced psoriatic skin lesions. In contrast, rapamycin suppressed the elevated effects of TCDD on P65 mRNA expression and phosphorylation of the P65 protein. (Figure 9A,B).

## 3. Discussion

The present in vivo study found that rapamycin ameliorated the TCDD-induced aggravation of IMQ-induced skin inflammation in mice. On the other hand, the environmental pollutant TCDD, known as dioxin, deteriorated IMQ-induced psoriatic-like dermatitis in mice. TCDD upregulated AHR and suppressed autophagy-related factor expression in IMQ-induced psoriatic mouse skin. Rapamycin downregulated AHR expression and increased autophagy-related factor expression in IMQ-induced psoriatic mouse skin. We previously demonstrated that the AHR agonist TCDD induced inflammation through autophagy inhibition using an in vitro psoriasis keratinocyte model and ex vivo psoriatic skin tissue study, and these findings are consistent with those noted in this study [31].

The complexity of psoriasis pathogenesis is slowly unveiled, and the role of environmental factors has been offered as an explanation for part of its pathophysiological intricacy. The role of lifestyle and the environment is suggested as modulators of the efficacy of psoriasis treatments. Abuse of alcohol, cannabis, and tobacco affects the treatment response of biologics in psoriasis [32]. Evidence regarding the effects of the environmental contaminant TCDD via AHR activation on the pathogenesis of psoriasis has accumulated [31,33]. AHR is a ligand-dependent transcription factor that plays a key physiological role in the maintenance of homeostasis in various organs and tissues and also influences pathological xenobiotic toxic effects [34]. This study showed that TCDD aggravated IMQ-induced skin inflammation in mice, consistent with other studies [33]. In keratinocytes, environmental contaminants, such as benzopyrene, increased the secretion of proinflammatory cytokines via the activation of AHR [35]. The present study found that TCDD induced AHR activation and subsequently increased the production of proinflammatory cytokines, including TNF-α, IL-6, IL-17A, IL-22, and IL-23, in IMQ-induced psoriatic lesional skin of mice. Previous studies have reported that AHR activation via exogenous or endogenous ligands induces Th17 cell development and increased IL-17A and IL-17F expressions [36]. Furthermore, AHR controls Th17 differentiation in a ligand-specific manner [37]. Cochez et al. reported that AHR is required for IL-22 production by Th17 cells in an imiquimod-induced psoriasis mouse model [38]. Given the key role of the TNF-α/IL-23/IL-17A axis in the pathogenesis of psoriasis, it could be hypothesized that polycyclic aromatic hydrocarbons (PAHs), such as TCDD, via AHR activation have a potential impact on the development or aggravation of the disease.

Studies have indicated that autophagy dysfunction in keratinocytes is involved in psoriatic inflammation [26,39]. We previously indicated that AHR activation via TCDD suppressed keratinocyte autophagy, inducing psoriatic inflammation in in vitro and ex vivo studies [31]. The present study demonstrated that in an IMQ-induced psoriatic mouse model, AHR activation through TCDD inhibited autophagy-related factor expression in lesional skin and subsequently aggravated skin inflammation clinically as well as molecular changes. These data suggest a possible role for AHR activation and autophagy control via TCDD in the inflammatory process of psoriasis.

The role of inflammation-dependent mTOR activation has been revealed to be a psoriatic pathomechanism. Inflammatory cytokines induce aberrant mTOR activity, which leads to enhanced epidermal proliferation [40]. Rapamycin is an inhibitor of mTORC1, requiring binding to FKBP12, its intracellular receptor [41]. Rapamycin is known for its antiproliferative and immunosuppressive properties on lymphoid cells [42]. Moreover, anticancer effects of rapamycin have been reported [43]. In particular, in a few small studies, rapamycin was systemically investigated for its antipsoriatic activity in psoriasis patients [44,45,46]. In this study, topical rapamycin improved skin inflammation in IMQ-induced psoriatic mice, which is consistent with a previous study [47]. Bürger et al. reported that the efficacy of rapamycin in an IMQ-induced psoriasis mouse model was related to reducing mTORC1 activation and restoring the expression and distribution of epidermal differentiation markers [47]. In the present study, we found that the mTOR inhibitor rapamycin suppressed the expression of proinflammatory cytokines, such as TNF-α, IL-6, IL-17A, IL-22, and IL-23, in IMQ-induced psoriatic dermatitis lesions. These results are consistent with those of a previous study showing the anti-inflammatory effects of rapamycin on keratinocytes [48]. Therefore, rapamycin might have an antipsoriatic effect by reducing inflammatory cytokines.

This study demonstrated that the mTOR inhibitor rapamycin could ameliorate psoriatic inflammation by inducing autophagy. Recently, the interaction between autophagy and the NF-κB signaling pathway or the link to autophagy in regulating oxidative stress has been shown in various cell or disease models [49,50,51]. Several in vitro studies have reported that autophagy suppression is related to the development and progression of psoriatic skin pathology [26,31]. We found that topical rapamycin restored autophagy flux and antioxidant-related enzyme activity and decreased NF-κB phosphorylation and oxidative stress markers in an in vivo psoriatic mouse model. Another mechanism of rapamycin action through mTOR inhibition might contribute to the diminution of IMQ-induced psoriatic inflammation by the regulation of immune cells’ energy metabolism, thereby controlling their function and differentiation. Dysregulated mTORC1 signaling was found in regulatory T cells and peripheral blood mononuclear cells (PBMCs) of psoriasis patients [52,53]. These molecular effects could account for the antipsoriatic impacts of rapamycin on TCDD-induced aggravated IMQ-induced psoriatic skin.

Rapamycin is a cytochrome P450 (CYP) 3A4 inducer. CYPs are the major enzymes involved in xenobiotic metabolism [54]. Although there have only been a few published reports that have identified the human CYP isoforms responsible for TCDD metabolism, TCDD was metabolized by CYP1A1 and CYP1A2 in rats [55]. Recently, it has been reported that IMQ is metabolized predominantly by CYP1A1 and CYP1A2 in human keratinocytes and the mouse liver [56]. Altogether, the metabolic effects of CYP3A4 induced by rapamycin on TCDD and IMQ may be relatively low.

As the largest secondary lymphoid organ in the immune system, the spleen participates in the process of systemic inflammatory immunoreactions [57]. In our study, we found that the weight and size of the spleen were significantly increased in the IMQ group compared with controls, consistent with the previous result [58]. TCDD application led to an increase in spleen size and weight in IMQ-induced psoriatic mice. In contrast, rapamycin reduced spleen size and weight in IMQ-induced psoriatic mice and TCDD-treated IMQ-induced psoriatic mice. These results suggest that IMQ and TCDD treatments contributed to the activation of systemic inflammatory immune responses as well as local skin reaction. However, rapamycin may be involved in the inhibition of inflammatory immune responses.

The imiquimod-induced psoriasis model showed several limitations, such as the relatively nonspecific nature of the induced skin inflammation, nonstandardized protocols for topical application, and unsuitability for chronic use [59].

## 4. Materials and Methods

### 4.1. Mice and Treatment

Specific pathogen-free female C57BL/6 mice (8 weeks old, 18–22 g) were obtained from Central Lab Animals (Central Lab. Animal Inc., Seoul, Korea). These mice were reared in a temperature-controlled room at 24 ± 2 ℃ and 55% ± 15% humidity with 12 h light and dark cycles. All experiments were performed under protocols approved by the Animal Research Ethics Board of Hallym University (HMC-2019-3-1202-45).

Mice were adapted to the laboratory for 1 week and then shaved on the back and right ear. One day later, 62.5 mg Aldara cream (Meda Pharmaceuticals, Vienna, Austria) was topically administered to the mice daily on shaved skin for 4 constitutive days, except for the mice in the control group. Aldara^®^ (imiquimod, IMQ) 5% cream (Meda Pharmaceuticals, Vienna, Austria) was used to induce psoriatic skin changes in the mice.

Experimental groups included five regimens: vehicle (50 mg petrolatum), IMQ (62.5 mg Aldara^®^ cream) and TCDD (Sigma-Aldrich, St. Louis, MO, USA) (100 nM) + IMQ (62.5 mg Aldara^®^ cream), rapamycin (Sigma-Aldrich, St. Louis, MO, USA) (5 mg/mL) + IMQ (62.5 mg Aldara^®^ cream), and TCDD (100 nM) + rapamycin (5 mg/mL) + IMQ (62.5 mg Aldara^®^ cream). To measure the severity of clinical inflammation on the back, a scoring system similar to the human Psoriasis Area and Severity Index (PASI) score was used [60]. In brief, erythema, scales, and thickness of the skin were scored “blindly.” The individual score was scaled from 0 to 3 as follows: 0: none; 1: slight; 2: moderate; and 3: severe. The single scores were summed, resulting in a maximal total score of 9. Skin thickness was assessed by measuring the double skin-fold thickness (DSFT) of the ears of the mice with a spring-loaded engineer micrometer (Mitutoyo, Kawasaki, Japan) throughout the experiment. Mice were euthanized with an overdose of isoflurane, and all efforts were made to minimize suffering.

### 4.2. Western Blot Analyses

The mouse skin tissues were harvested into PRO-PREP™ lysis buffer (iNtRON, Seoul, Korea) containing a protease inhibitor cocktail (Roche Diagnostics, Mannheim, Germany). We used copper (II) sulfate solution in bicinchoninic acid solution (Sigma-Aldrich, St. Louis, MO, USA) to measure the protein concentrations. Equal amounts of protein (20 µg) were separated by 10% sodium dodecyl sulfate–polyacrylamide gel electrophoresis, transferred to enhanced chemiluminescence (ECL) nitrocellulose membranes (GE Healthcare, Chicago, IL, USA), and then blocked for 1 h with 5% skim milk in tris-buffered saline with 0.1% TWEEN^®^ 20. The membranes were incubated overnight at 4 °C with rabbit anti-AHR (1:1000, Abcam, Cambridge, MA, USA), rabbit anti-CYP1A1 (1:1000, Abcam, Cambridge, MA, USA), rabbit anti-P62 (1:1000, Abcam, Cambridge, MA, USA), rabbit anti-ATG5 (1:1000, Abcam, Cambridge, MA, USA), rabbit anti-LC3 (1:1000, Abcam, Cambridge, MA, USA), rabbit anti-Beclin1 (1:1000, Novusbio, Centennial, CO, USA), rabbit anti-NOX2 (1:1000, Abcam, Cambridge, MA, USA), rabbit anti-NOX4 (1:1000, Abcam, Cambridge, MA, USA), rabbit anti-Nrf2 (1:1000, Abcam, Cambridge, MA, USA), and rabbit anti-phospho P65 (1:1000, Abcam, Cambridge, MA, USA). The primary antibodies were detected with horseradish peroxidase-conjugated goat anti-rabbit (1:1000, Abcam, Cambridge, MA, USA) IgG secondary antibodies. We visualized the protein bands with a LuminoGraph II (Atto, Tokyo, Japan). Densitometric analysis of the blots was performed using ImageJ software (National Institutes of Health).

### 4.3. Quantitative Reverse Transcription Polymerase Chain Reaction (qPCR)

According to the manufacturer’s instructions, we extracted total RNA using the RNeasy^®^ Plus Mini Kit (Qiagen, Hilden, Germany). We used a Transcriptor First Strand cDNA Synthesis Kit (Roche Applied Science, Mannheim, Germany) to synthesize cDNA from total RNA (1 µg). We performed quantitative reverse transcriptase polymerase chain reaction (qPCR) three times using the TaqMan™ Master Mix and Real-Time PCR system (Applied Biosystems, Foster City, CA, USA). We normalized the mRNA levels of AHR (TaqMan Assay ID Mm00478932_m1), CYP1A1 (TaqMan Assay ID Mm00487218_m1), LC3 (TaqMan Assay ID Mm00458724_m1), Beclin1 (TaqMan Assay ID Mm01265461_m1), ATG5 (TaqMan Assay ID Mm01187303_m1), TNF-α (TaqMan Assay ID Mm00443258_m1), IL-6 (TaqMan Assay ID Mm00446190_m1), IL-17A (TaqMan Assay ID Mm00439619_m1), IL-22 (TaqMan Assay ID Mm00444241_m1), IL-23 (TaqMan Assay ID Mm00518954_m1), NOX2 (TaqMan Assay ID Mm01287743_m1), NOX4 (TaqMan Assay ID Mm00479246_m1), Nrf2 (TaqMan Assay ID Mm00477784_m1), and P65/NF-κb (TaqMan Assay ID Mm01310735_m1) to that of glyceraldehyde-3-phosphate dehydrogenase (TaqMan Assay ID Mm99999915_m1). Relative quantification was performed using a LightCycler^®^ 96 Instrument (Roche Diagnostics, Mannheim, Germany).

### 4.4. Immunohistochemistry

Immunohistochemistry was performed in 10% formalin-fixed, paraffin-embedded tissues. The dissected tissues were washed several times with distilled water and treated with 1% sodium borohydride for 1 h to remove any residual fixatives. The tissues were pretreated with 3% hydrogen peroxide solution for 10 min, washed with distilled water, and cultivated for 5 min with 1 TBST (tris-buffered saline 0.1% Tween 20). To prevent nonspecific reactions, the tissues were treated at room temperature at approximately 20–22 °C with normal goat serum (Vector Laboratories, Burlingame, CA, USA). Then, the tissues were cultivated overnight with rabbit anti-AHR (1:300; Abcam, Cambridge, MA, USA), rabbit anti-CYP1A1 (1:300; Abcam, Cambridge, MA, USA), rabbit anti-P62 (1:300; Abcam, Cambridge, MA, USA), rabbit anti-ATG5 (1:300; Abcam, Cambridge, MA, USA), rabbit anti-LC3 (1:300; Abcam, Cambridge, MA, USA), and rabbit anti-Beclin1 (1:300; Abcam, Cambridge, MA, USA). Tissues were washed with 1X TBST and incubated for 30 min at room temperature with biotinylated secondary antibody solution from the Dako REAL EnVision Detection System (Dako, Glostrup, Denmark). Next, tissues were washed with distilled water, counterstained with hematoxylin (Sigma-Aldrich, St. Louis, MO, USA), dehydrated and clarified using a conventional method, and prepared for Leica microsystems DFi8 LASX software light microscopy (Leica, Wetzlar, Germany). The level of staining was semiquantitatively analyzed using LAS X software (Leica, Wetzlar, Germany). The results are expressed as the mean optical density (±standard deviation) of six different digital images. The epidermis thickness was measured from the bottom of the rete ridge to the bottom of the stratum corneum using a calibrated ruler. The mean epidermal thickness was calculated from the measurements in four fields at ×100 magnification [61].

### 4.5. The Measurement of Spleen in Mice

The spleen was isolated from mice, and a photograph was taken before being calculated. Splenomegaly was evaluated by measuring the ratio of the weight of the spleen to the bodyweight [62].

### 4.6. Statistical Analyses

We conducted statistical analyses with GraphPad Prism version 5.01 (GraphPad Software, San Diego, CA, USA). The clinical severity parameters (total score, thickness, scales, erythema, and ear thickness) were analyzed using two-way repeated measures analysis of variance (ANOVA) with Tukey’s post hoc test, with group and time as grouping variables. Differences in the epidermal thickness; spleen weight index; mRNA expression levels of AHR, CYP1A1, and autophagy-related factor; proinflammatory cytokine mRNA expression levels; and mRNA expression levels of oxidative stress-related factors among the groups were analyzed using one-way repeated measures ANOVA with Dunnett’s post hoc test. Data are presented as mean ± standard deviation (SD). Differences were considered statistically significant at *p* < 0.05.

## 5. Conclusions

In summary, we demonstrated that rapamycin alleviated the aggravated dermatitis induced by TCDD in mice with imiquimod-induced psoriasis-like dermatitis. Rapamycin restored TCDD-induced autophagy suppression. In addition, rapamycin suppressed the TCDD-induced elevated effects on oxidative stress and inflammatory response in skin lesions in a psoriatic mouse model. These results indicate the relevance of environmental pollutants, such as dioxin, in the pathogenesis of psoriasis and the promiscuous possibility of using the autophagy inducer rapamycin as a novel therapeutic target for psoriasis. The novel mTOR inhibitors may be simultaneously beneficial for several psoriasis-related inflammatory and noninflammatory comorbidities.

## Data Availability

The data presented in this study are available on request from the corresponding author.

## Abbreviations

AHR	aryl hydrocarbon receptor
TCDD	2,3,7,8-tetrachlorodibenzo-p-dioxin
NF-κB	nuclear factor-kappaB
Rapa	rapamycin
mTOR	mechanistic target of rapamycin
mTORC1	mechanistic target of rapamycin complex 1
IMQ	imiquimod
H&E	hematoxylin and eosin
ECL	enhanced chemiluminescence
ANOVA	analysis of variance
TBST	tris-buffered saline 0.1% Tween 20
CYP1A1	cytochrome P450 family 1 subfamily A member 1
ATG5	autophagy related 5
LC3	microtubule-associated proteins 1A/1B light chain 3B
NOX2	NADPH oxidase 2
NOX4	NADPH oxidase 4
Nrf2	nuclear factor-erythroid 2-related factor 2
PASI	Psoriasis Area and Severity Index
DSFT	double skin-fold thickness
QPCR	quantitative reverse transcription polymerase chain reaction
GAPDH	glyceraldehyde 3-phosphate dehydrogenase
IL-1 β	interleukin-1β
IL-6	interleukin-6
IL-17A	interleukin-17A
IL-17F	interleukin-17F
TNF- α	transforming growth factor-α
